# Effects of Virtual Pedagogical Agents and Instructional Content Type on Learners’ Experience in Online Learning: Evidence from Behavioral, Eye-Tracking, and EEG Data

**DOI:** 10.3390/bs16091691

**Published:** 2026-09-19

**Authors:** Shasha Liu, Xiaoqi Shen, Tiansheng Xia

**Affiliations:** 1 School of Literature, History, and Media, Xuchang University, Xuchang 461000, China; 2 School of Art and Design, Guangdong University of Technology, Guangzhou 510006, China

**Keywords:** online learning, virtual pedagogical agent, anthropomorphism, learning outcomes, eye tracking, electroencephalography

## Abstract

Online learning has become an important means of acquiring knowledge. Virtual pedagogical agents have gradually evolved from auxiliary tools into intelligent instructional media capable of affective interaction. However, how these agents affect learning outcomes remains unclear. Integrating social agency theory and cognitive load theory, this study developed a model in which virtual pedagogical agent type was the independent variable; learning experience was the dependent variable; psychological distance, emotional attachment, and attentional engagement were mediators; and instructional content type was the moderator. A 2 (virtual pedagogical agent: human-like vs. non-human) × 2 (instructional content type: traditional knowledge vs. interest-oriented popular science) mixed design was used. Questionnaires, eye tracking, and electroencephalography (EEG) were combined to assess the learning processes of 40 university students. The results showed that the interaction between agent type and instructional content type significantly affected learning experience. Specifically, in learning contexts featuring traditional knowledge content, human-like virtual agents were associated with higher learning satisfaction, greater emotional attachment, and lower psychological distance. In contrast, during interest-oriented content learning, non-human agents achieved superior knowledge recall scores, along with higher learning satisfaction, stronger emotional attachment, and lower psychological distance. Furthermore, eye-tracking data showed that attentional allocation was also affected by this combination. EEG data showed that FCz spectral power varied with it as well, however, because power rose simultaneously in theta, alpha, beta, and gamma; this broadband pattern is not an unambiguous index of deeper processing and may partly reflect a general increase in signal amplitude. In addition, the moderated-mediation analysis revealed that psychological distance and emotional attachment mediated the relationship between virtual-agent type and learning experience, while knowledge type moderated these mediating paths. The practical implications for online learning are discussed.

## 1. Introduction

Rapid advances in artificial intelligence and virtual digital technologies have created new pathways for the digital transformation of higher education. These advances have reshaped educational cognition and moved virtual characters from passive support tools toward genuine instructional agents. Virtual characters are thus being repositioned from tools to educational partners. Nevertheless, their widespread adoption has not consistently improved the learning experience. Their effectiveness varies substantially across contexts (Schroeder et al., 2025). Virtual pedagogical agents may affect psychological distance (Franke & Groeppel-Klein, 2024), emotional attachment (A. Zhang & Rau, 2023), attentional engagement, and other factors, thereby producing different learning experiences. Instructional content type is also an important contextual factor (X. Xu et al., 2026), and it may condition the effects of virtual pedagogical agents. Identifying the mechanisms and boundary conditions of these effects is therefore important for developing virtual pedagogical agents for online learning and improving online learning outcomes.

In recent years, researchers have examined the effectiveness and influence of virtual pedagogical agents from several perspectives. This attention reflects persistent challenges in online learning, including low engagement, distraction, and limited personalization. The appearance and behavior of virtual pedagogical agents can influence the acquisition of factual knowledge (Petersen et al., 2021). Herbert and Dołżycka (2022) argued that such agents may primarily affect learner motivation and enjoyment of online learning. Xia et al. (2024) found that avatar-based methods significantly increased positive emotions and satisfaction compared with camera-based monitoring. As a pedagogical method, behavioral modeling by virtual pedagogical agents can also improve motivation, particularly self-efficacy, and learning outcomes such as declarative knowledge and task performance (Fountoukidou et al., 2019). These agents may increase positive emotion, intrinsic motivation, and learning performance, and their characteristics may moderate the effects of affective pedagogical agents (Y. Wang et al., 2023b). However, some studies have found that virtual characters are less effective than human teachers and yield lower knowledge-recall scores (Rativa et al., 2021). Although perceived humanness and attractiveness influenced students’ perceptions, these characteristics did not significantly affect learning outcomes (Schroeder et al., 2017) or knowledge acquisition (S. Zhang et al., 2024). Virtual pedagogical agents may also increase extraneous cognitive load (Y. Wang et al., 2023a), divert attention from essential information, and consequently produce only weak learning benefits. In addition, instructional content type is another basic variable that influences pedagogical strategy and media selection. Research on multimedia learning and educational technology suggests that an effective match between instructional media and content is central to improved learning (Schroeder et al., 2025). Intelligent learning environments support adaptive instructional strategies and personalized learning (Peng et al., 2019).

Taken together, these strands of work leave two questions open. The first concerns whether human-likeness is inherently beneficial. Some studies report that more human-like agents raise social presence, trust, and satisfaction, whereas others report null effects on learning outcomes or discomfort of the kind associated with the uncanny valley. The second concerns the level at which agent effects should be theorised. Most studies treat the agent as a fixed property of the learning environment and ask whether it helps, rather than asking what it helps with. Much research that does consider content has generally used it as a control variable, so the possibility that the sign of an agent effect depends on the material being taught has rarely been tested directly. This study builds on that work by treating agent effectiveness as conditional rather than absolute, and it departs from it in three ways. It models instructional content type as a moderator rather than as a nuisance variable, and it therefore predicts a crossover interaction rather than an advantage for either agent. It combines self-report, eye-tracking, and electroencephalographic (EEG) measures within a single learning session, so that the affective, attentional, and neurophysiological correlates of the same learning episode can be examined together rather than inferred across studies. It also specifies and tests the pathway linking the combination of agent and content to learning satisfaction, through psychological distance and emotional attachment, instead of documenting an effect without a mechanism.

## 2. Literature Review and Hypotheses

This section reviews four bodies of literature, each corresponding to one element of the model tested here. It begins with the two theoretical frameworks that guide the study, social agency theory and cognitive load theory, because together they specify both why social cues in an agent can support learning and why the same cues can interfere with it. It then reviews work on virtual pedagogical agents and learning experience, which supplies the operational distinction between human-like and non-human agents and thus defines the independent variable. The following subsection reviews psychological distance, emotional attachment, and attentional engagement, the three candidate mechanisms through which agent effects are thought to operate, which serve as the mediators. The final subsection reviews research on instructional content, which motivates treating content type as a boundary condition and hence as the moderator.

### 2.1. Social Agency Theory and Cognitive Load Theory

Social agency theory (SAT) provides a central social-cognitive perspective for explaining how virtual pedagogical agents can facilitate learning. It is grounded in the media equation proposed by Reeves and Nass (1996), which holds that people often apply interpersonal social rules to computers and other media without conscious awareness. This paradigm was later introduced into multimedia learning to evaluate the effectiveness and utility of simulated interpersonal connections in technology-mediated settings (Moreno et al., 2001). SAT proposes that verbal cues, such as a human-like voice and personalized dialogue, and visual cues, such as an anthropomorphic appearance, gaze, and gestures, can elicit a social response from learners. This response establishes a virtual partnership that affects learning through two related pathways. One pathway is socioemotional: social cues reduce psychological distance between the learner and the computer, strengthen social presence, and increase interest and motivation (Moreno et al., 2001). The other way is a deep cognitive pathway. Once a collaborative relationship is established, learners may implicitly apply the cooperative principles of human communication. They assume that the agent’s explanation is cooperative and invest greater cognitive effort in selecting, organizing, and integrating information, thereby improving retention and transfer (Mayer et al., 2003). SAT has been widely used to explain the role of pedagogical agents and other virtual humans, including the effects of voice (Craig & Schroeder, 2017), appearance (Haake & Gulz, 2009), instructional style, and gestures (Baylor & Kim, 2009). Cognitive neuroscience also provides physiological support for SAT. Using functional near-infrared spectroscopy, W. Li et al. (2022) found stronger activation in brain regions related to social cognition, including the superior temporal gyrus and inferior frontal gyrus, among students who watched videos featuring embodied agents with gestures, gaze, and movement. This finding directly supports the proposition that an agent’s social cues activate deeper social-cognitive processing.

Cognitive load theory (CLT), proposed by Sweller (1988), defines cognitive load as the mental energy required to process given information and focuses on how to support problem solving and effective learning (Schnotz & Kürschner, 2007). The theory distinguishes intrinsic, extraneous, and germane cognitive load (Sweller, 1988). Learners must manage all three forms when they encounter new information. Intrinsic cognitive load depends on the difficulty of the content and the learner’s prior knowledge. Inappropriate instructional arrangements, including poor task presentation and irrelevant stimuli, increase extraneous load. Germane cognitive load reflects the cognitive effort invested in acquiring knowledge (Paas & Van Merriënboer, 2020). Common empirical measures include subjective reports, such as self-rating scales; task-performance measures, such as completion time and accuracy; and physiological measures, such as heart rate, eye movements, and EEG activity.

Studies of online learning often combine SAT and CLT to examine new theoretical models and learning technologies. For example, Craig and Schroeder (2017) used both theories to investigate the effects of different voice types on learning. SAT predicts that integrating social cues into multimedia messages can promote deeper learning relative to conditions without such cues (Mayer et al., 2003). Social cues can create affinity and a personalized experience, thereby reducing psychological distance. When a virtual pedagogical agent displays appealing, content-congruent personal characteristics with positive valence, learners are more likely to experience social presence (Domagk, 2010), which can motivate learning.

CLT attributes extraneous cognitive load to the way instructional materials are presented, and poorly designed instruction therefore imposes unnecessary demands on learners (Kirschner, 2002). For example, an inappropriate virtual pedagogical agent may introduce irrelevant visual or auditory distractions and divide attention. Learners may need additional time and effort to interpret the agent’s social cues. This demand can increase psychological distance and make the agent feel unfamiliar or disconnected from the learning context. SAT and CLT explain the effects of virtual agents through two complementary mechanisms: social-cognitive activation and cognitive-resource allocation. SAT emphasizes appropriate social cues that stimulate motivation and emotional connection. CLT emphasizes the control of irrelevant information to maintain efficient processing. Together, the theories suggest that effective agent use does not depend simply on adding or removing social characteristics. Instead, the pedagogical agent’s appearance must be adapted to learners’ cognitive and emotional demands for different instructional contexts. When agent characteristics align with the complexity and affective appeal of the content, social cues can support learning without creating unnecessary interference. This integrated perspective provides the theoretical basis for examining the effect of virtual pedagogical agents and instructional content.

Two commitments of these frameworks are carried forward into the present study. From SAT we take the claim that social cues are processed automatically and that their effect is graded rather than binary: an agent does not simply possess or lack social cues; it presents cues of a particular kind, and learners respond to the relation between those cues and the situation. This claim licenses the prediction that agent type will interact with, rather than add to, content type. From CLT we take the claim that any cue irrelevant to the schema currently being constructed consumes working-memory capacity that would otherwise be available for learning. This claim specifies the cost side of the interaction and identifies where a poorly suited agent should be most damaging, namely for content that already imposes high intrinsic load.

### 2.2. Virtual Pedagogical Agents and Learning Experience

Virtual pedagogical agents are static or animated anthropomorphic interfaces used in e-learning environments to support instructional goals. They often display human-like characteristics, including emotion, responsiveness, and language ability. They may appear as humans or as talking animals, cartoons, insects, or other characters (Veletsianos, 2010). More specifically, a virtual pedagogical agent is any visually present character designed to facilitate learning in an instructional environment (Craig & Schroeder, 2017). Such agents can support learning by signaling important information, delivering motivational messages, or acting as facilitators. In the present study, the virtual pedagogical agent served as the course instructor.

Based on visual characteristics, Yan et al. (2024) classified virtual characters as simulated-real human characters, which are photorealistic and almost indistinguishable from humans; animated human characters, which depict humans in an animated style; and non-human characters, which represent animals, inanimate objects, or otherworldly beings. Educational research has more often compared particular virtual characters, such as robotic animals (Rativa et al., 2021), robots (J. Li et al., 2016), and animated humans (W. Li et al., 2022), with real human teachers.

Learning experience refers to learners’ perceptions and gains throughout the learning process. It includes perceived learning and learning performance. Learning satisfaction and cognitive load are widely used measures of perceived learning. Learning satisfaction reflects learners’ affective responses and their satisfaction with the learning process and outcomes. It includes emotional experience and interest in the learning materials (Goh et al., 2017). Satisfaction is shaped by cognitive processes, emotional state, and motivation. Cognitive load is the total amount of cognitive resources required to complete a task (Paas et al., 2010). It reflects brain activity related to working memory, attention, and cognitive control. Cognitive load comprises intrinsic, extraneous, and germane load. These forms reflect task difficulty, the learner’s prior knowledge, and the presentation of instructional materials (Paas et al., 2010). Because cognitive load is a multidimensional construct whose components cannot be read directly from a single physiological index, the present study used EEG spectral power as an index of cognitive processing rather than to quantify cognitive load as such. Learning performance is commonly assessed through knowledge recall, which directly indicates the learner’s ability to retrieve and apply information after processing it.

Virtual pedagogical agents may significantly affect the learning experience. J. Li et al. (2016) suggested that well-designed virtual characters can be viable alternatives to human teachers. Polat et al. (2025) found that human-like virtual teachers enhanced social presence, engagement, and satisfaction in knowledge-learning contexts, with effects comparable to those of human teachers. Xia et al. (2026) found that anthropomorphized strategies enhanced learners’ social presence and evoked positive emotions, which in turn improved learning outcomes. Other studies have reported that robotic-animal pedagogical agents are less effective than agents with animal or human appearances (Rativa et al., 2021) and may even produce discomfort similar to the uncanny valley effect. Despite growing research on virtual characters, the specific educational effects of human-like and non-human virtual pedagogical agents remain insufficiently understood. Domagk (2010) divided virtual agents into human agents, such as recordings of real people or human-like characters, and non-human agents, such as animals or fictional characters. Recent research has called for more empirical evidence on human-like and fictional, non-human agents in digitally enhanced learning (Dai et al., 2024). Human-like agents simulate real people and have highly realistic features. Their human appearance and behavior may improve interactivity and credibility (Franke & Groeppel-Klein, 2024), which can foster trust and engagement. Non-human agents represent animals, inanimate objects, or otherworldly beings. Virtual animals and pets in particular (Chen et al., 2011) may reduce uncanny-valley discomfort and make learning more enjoyable and appealing (Yan et al., 2024).

Following SAT, because both agents supply social cues, any difference between them cannot be attributed to the presence or absence of social agency, only to the kind of social schema each activates. CLT supplies the competing consideration that a visually richer human-like agent may consume additional visual-channel capacity without adding instructionally relevant information. Accordingly, Hypothesis 1 (H1) was proposed: Virtual pedagogical agent type significantly affects learners’ learning experience.

### 2.3. Mediating Roles of Psychological Distance, Emotional Attachment, and Attentional Engagement

Psychological distance is the subjective experience that a person or object is close to or distant from the self (Trope & Liberman, 2010). It includes social, temporal, spatial, and hypothetical distance. Social presence theory proposes that digital interfaces influence perceived psychological distance by providing different levels of social cues and interactivity. They can thereby strengthen or weaken social presence (Biocca et al., 2003). Anthropomorphic virtual pedagogical agents can increase perceived similarity and reduce psychological distance, which improves message credibility and persuasiveness (Ahn et al., 2021). Franke and Groeppel-Klein (2024) identified psychological distance as a potential mechanism underlying perceptions of virtual characters’ credibility and novelty. Animated characters produced greater psychological distance than human-like characters, suggesting lower social presence. These two lines of evidence point in opposite directions regarding how agent human-likeness relates to social presence, which suggests that the direction of the effect is not fixed but context-dependent—an issue the present study addresses through the match between agent type and instructional content. In virtual settings such as social media, social presence has also been proposed as a key mechanism through which virtual influencers affect emotional attachment and benefit-seeking behavior (Yan et al., 2024). In that context, non-human virtual influencers produced greater social presence than human-like virtual influencers. In research on intelligent-agent design, social presence positively and significantly mediated the relationship between agent design and behavioral outcomes (A. Zhang & Rau, 2023). Thus, psychological distance can mediate the relationship between virtual-character design and behavior. When this distance is small, learners are more likely to trust and become emotionally attached to the agent, and to accept the knowledge it presents.

Because psychological distance is the proximal consequence of social cues in SAT terms, and because an agent perceived as distant requires additional interpretive effort, psychological distance should also track extraneous processing demands. Hypothesis 2 (H2) was proposed: Virtual pedagogical agents affect learners’ psychological distance, which in turn affects their online learning experience.

Emotional attachment is an affect-laden, target-specific bond between a person and an object. It comprises three interrelated components: affection, passion, and connection (Thomson et al., 2005) and helps sustain intimacy and closeness in meaningful relationships. In human–computer interaction, people may instinctively apply preconceptions when a machine initiates an interaction. They may view the machine as mechanical, impartial, unemotional, and distant, which can shape their behavior. Emotional attachment is therefore important in interactions with both humans and machines (S. Zhang et al., 2024). In online marketing, virtual influencers can provide additional signaling value, such as innovativeness, openness, or responsiveness to current trends, and thereby influence emotional attachment (Yan et al., 2024). In research on intelligent agents, strong anthropomorphism and a mentor role significantly and positively predict emotional attachment (A. Zhang & Rau, 2023). In education, an AI virtual instructor whom learners like or admire can increase motivation and foster positive learning emotions (Pataranutaporn et al., 2022). Emotional attachment can also support positive evaluations of the learning context (Özer et al., 2023). Liu et al. (2024) showed that emotional engagement is related to educational outcomes and plays an important role in predicting achievement and satisfaction. Carefully designed avatars may therefore strengthen emotional attachment (Schöbel et al., 2019) and improve learning outcomes.

Accordingly, Hypothesis 3 (H3) was proposed: Virtual pedagogical agents significantly affect online learning experience through emotional attachment.

Attention is a psychological state in which an individual selectively focuses on a particular object and sustains engagement with it. Multimedia-learning research suggests that the presence of an instructor in an instructional video has complex effects on attention. On the one hand, the instructor’s image may attract attention and create a split-attention effect (Ayres & Sweller, 2005), because learners must divide attention between the instructor and the presented content. Working-memory capacity may then be consumed by processes that do not support learning. On the other hand, J. Wang and Antonenko (2017) found that learners who could see the instructor reported greater satisfaction with both simple and difficult topics than learners who could not. They also invested less effort in difficult topics and recalled simple topics more effectively. These findings suggest that instructor presence may benefit learning through social or affective processes. In addition, instructor nonverbal behavior and its fit with instructional content and context influence learners’ attention (Davis, 2018). Virtual pedagogical agents may therefore help learners sustain attention and improve learning outcomes. Flow theory further suggests that highly focused attention can lead to a flow state and improve both perceived learning and performance (Shernoff et al., 2003).

Prior eye-tracking work has established where learners look when an instructor is visible, but has seldom linked gaze allocation to satisfaction within the same design. The present study extends it by treating fixation duration as a mediator rather than as a descriptive outcome. This is where CLT is most directly implicated, because the split-attention effect is a cognitive-load prediction and fixation duration in the agent area of interest provides a behavioral index of how much visual-channel capacity the agent consumes. SAT predicts the opposite sign, since looking at the agent may reflect social engagement rather than distraction. The two predictions are separated empirically by whether longer fixation on the agent accompanies higher or lower satisfaction. Accordingly, Hypothesis 4 (H4) was proposed: Virtual pedagogical agents affect attentional engagement, which in turn affects online learning experience.

### 2.4. Moderating Role of Instructional Content Type

The effects of virtual pedagogical agents may depend on instructional content type. Previous studies have sometimes treated content only as a control variable (Veletsianos, 2010) or classified it by knowledge structure rather than by the learner’s interaction with the instructional medium. Findings from related fields can clarify how content may condition agent effects. Research on online video platforms identifies perceived content value as a central determinant of user behavior and distinguishes knowledge content from entertainment content (Cao et al., 2024). This distinction shows that different value propositions produce different behavioral responses. Building on learners’ cognitive and motivational needs, we therefore propose a classification focused on two dimensions: the extent to which content requires the construction of cognitive schemas and the extent to which it must stimulate affective motivation. Goal-orientation theory distinguishes task (mastery) goals from performance goals (Dweck & Leggett, 1988). Task (mastery) goals emphasize understanding foundational knowledge and core concepts. Performance goals are more closely related to interest and achievement motivation and emphasize interest-driven exploration and practice. This distinction corresponds to two common orientations in online learning: systematic knowledge acquisition and interest-based enrichment. CLT and SAT suggest that systematic, mastery-oriented learning should prevent social cues from becoming a cognitive burden, whereas interest-oriented learning should use such cues to elicit affective engagement. The present study therefore operationalized instructional content type as traditional knowledge content and interest-oriented popular science content. Traditional knowledge content was mastery-oriented and emphasized foundational concepts, principles, and logical systems. Interest-oriented popular science content emphasized enjoyment and enrichment. This classification was specified on theoretical grounds before the experimental materials were selected, and the materials described in Section 3.3 were then chosen to instantiate the two categories, rather than the categories being derived post hoc from the materials that happened to be available. It used everyday examples or novel interdisciplinary and non-disciplinary topics to stimulate interest and was more closely aligned with performance goals.

Different virtual pedagogical agents may be suited to different types of online instructional content, and an appropriate match may increase their effectiveness (D. Xu & Wang, 2006). According to CLT (Sweller, 1994), contextually irrelevant information increases extraneous cognitive load because learners must attend to multiple schemas. Such irrelevance hinders learning by placing additional pressure on limited working-memory resources. When a pedagogical agent is highly relevant to the instructional content, working memory can be used more efficiently. Veletsianos (2010) showed that learners form stereotypes about pedagogical agents from their visual appearance, and these stereotypes affect perceived credibility and learning. Based on these stereotypes and learning preferences, human-like virtual pedagogical agents may be viewed as authoritative sources of knowledge and may therefore be more effective for traditional knowledge content. When learning interest-oriented popular science content, non-human agents may stimulate greater interest and engagement.

Accordingly, Hypothesis 5 (H5) was proposed: Instructional content type moderates the effects of virtual pedagogical agents. Specifically, the effects of agent type on psychological distance, emotional attachment, attentional engagement, and learning satisfaction vary by instructional content type.

## 3. Method

### 3.1. Participants

Forty university students volunteered to participate, including 11 men and 29 women. The gender distribution was 6 men and 14 women in the traditional knowledge group and 5 men and 15 women in the interest-oriented popular science group, with similar gender ratios between the two groups. All participants had experience with online learning and were physically and psychologically healthy. All participants were university students majoring in social-science-related fields with comparable educational levels. No participants reported conditions such as dyslexia or attention-deficit hyperactivity disorder (ADHD), or other traits that could impair information processing and learning abilities. None reported a psychological disorder, cold, fever, or other condition that could affect experimental performance. All had normal or corrected-to-normal vision, no color blindness or color-vision deficiency, and could clearly view the computer display. Participants read and signed written informed-consent forms and received a small cash payment after the experiment. The study was approved by the Academic Ethics Committee of Guangdong University of Technology (Approval No. GDUTXS20250015; approved on 21 February 2025). The sample size was determined by the throughput of the combined eye-tracking and EEG protocol, which requires approximately one hour of laboratory time per participant, and by the availability of the recording facility.

### 3.2. Experimental Design

The study used a 2 (virtual pedagogical agent: human-like vs. non-human) × 2 (instructional content type: traditional knowledge vs. interest-oriented popular science) mixed design. Instructional content type was the between-subjects factor, and agent type was the within-subjects factor. The 40 participants were randomly assigned to one of two content groups, with 20 participants per group. The dependent variables were learning satisfaction, psychological distance, emotional attachment, knowledge recall, attentional engagement, and FCz spectral power. Learning satisfaction, psychological distance, emotional attachment, and knowledge recall were assessed using questionnaires. Attentional engagement was measured through eye tracking, and spectral power was derived from the EEG recording at the FCz electrode, whose possible cognitive interpretation is discussed in Section 5.3.

SAT predicts effects on socioemotional appraisal, which the questionnaires capture, whereas CLT predicts effects on the allocation of limited processing capacity, which the eye-tracking and EEG measures index. The three classes of measures were chosen because the constructs of interest operate at different levels and no single method indexes all of them. Self-report questionnaires were used for psychological distance, emotional attachment, and learning satisfaction because these are subjective appraisals that are, by definition, accessible only to the learner, and because validated scales already exist for each construct (Fryer et al., 2019; Jiménez & Voss, 2014). Eye tracking was used because attentional allocation is not reliably available to introspection and because fixation duration within defined areas of interest is an established online index of where processing capacity is directed during multimedia learning; it has been used for precisely this purpose in studies of instructor presence and instructor gesture (Davis, 2018; J. Wang & Antonenko, 2017). EEG was used because it provides a continuous, non-intrusive record of cortical activity throughout viewing, at a temporal resolution that neither self-report nor gaze can match. This combination provides more explanatory evidence for investigating complex learning processes by leveraging multisource data (Xia et al., 2026).

Learning satisfaction and knowledge recall were treated as the primary outcomes, corresponding to the perceived-learning and performance components of learning experience, while psychological distance, emotional attachment, fixation duration, and FCz spectral power were treated as secondary outcomes serving the mediation model. No formal correction for multiple comparisons was applied. The tests reported are not independent examinations of separate questions but a set of related tests of one predicted interaction pattern, and the four frequency bands were analyzed band by band because they are not interchangeable indicators of the same process. Nevertheless, the number of tests reported raises the probability of at least one Type I error, and individual *p* values close to the conventional threshold should be read with that in mind. The interpretation offered in Section 5 therefore rests on the consistency of the interaction across independent measures rather than on any single test.

### 3.3. Experimental Materials

The experimental materials comprised four sets of instructional videos: traditional knowledge content presented by a human-like agent, interest-oriented popular science content presented by a human-like agent, traditional knowledge content presented by a non-human agent, and interest-oriented popular science content presented by a non-human agent. The materials were selected and produced as follows.

First, videos were selected for the two content categories. Traditional knowledge content and interest-oriented popular science content represented learning contexts with different cognitive-processing structures and motivational demands. The following selection criteria were developed from the literature: the content had to fit the category definition, cover a foundational course topic, have been used in previous research, and last 10–15 min. Two videos on applications of nanotechnology were selected as traditional knowledge content from the Chinese University MOOC platform. Two popular science videos on cat coat color were selected as interest-oriented content from the Bilibili platform. The nanotechnology material was chosen because it is a foundational topic within an accredited university course, requires the construction of a hierarchical conceptual schema, and presupposes no prior interest on the learner’s part. The cat coat color material was chosen because it is an everyday, visually concrete topic that is nevertheless governed by a formal system of genetic rules, so that it is genuinely instructional rather than merely entertaining, and because material on this topic is among the most widely viewed popular science content in its category on the platform.

Second, the two agent types were created. Agent images were generated on online platforms such as Jimeng (Version 3.0) and Doubao (Version 1.52.9), and voices were synchronized using CapCut (Version 8.7.0). 

**Figure 1:**
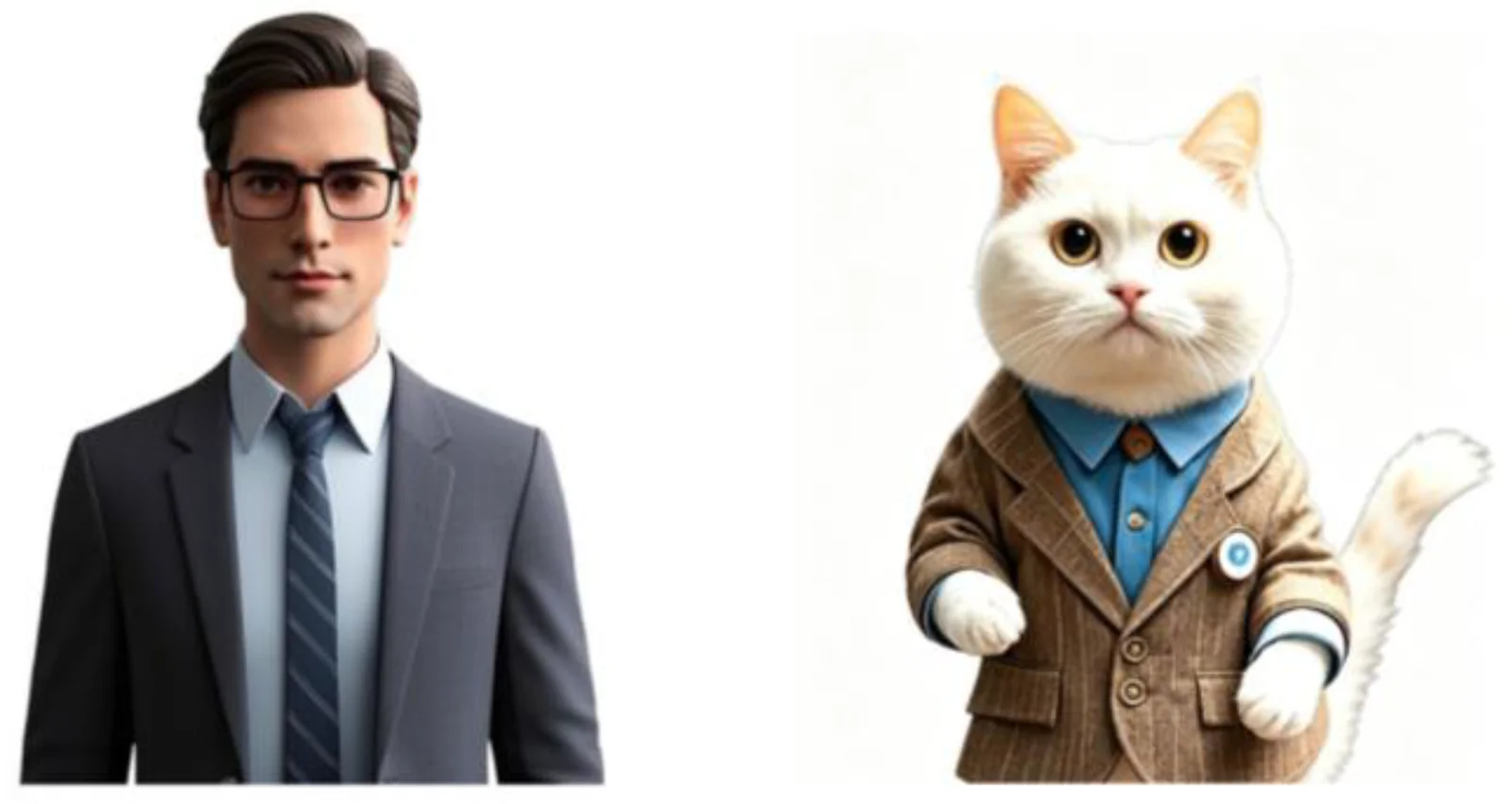
Two virtual pedagogical agents: human-like agent (**left**); non-human agent (**right**).

Drawing on previous research on virtual instructors, we designed the human-like agent to resemble a real teacher and to have an approachable, composed, and formal appearance (Kogan et al., 2018). The agent wore glasses and formal clothing such as a suit or shirt (Pi et al., 2022). The non-human agent was an AI-generated animal rendered in a realistic style. A familiar animal, a cat, was given anthropomorphic features. Clothing was kept as similar as possible across the two agents. The human-like agent used an AI-generated voice intended to simulate a real person, whereas the non-human agent used an AI-generated animated voice. The script, speech rate, delivery, and language style were held constant across the two agents, and both voices were synthesised on the same platform. The voices were not, however, pretested or normed for naturalness, emotional tone, attractiveness, or perceived human-likeness. The two agents are shown in Figure 1.

Finally, the source videos and virtual agents were edited into the experimental learning videos using CapCut. Irrelevant elements were removed, and consistent subtitles were added. Each agent appeared on the right side of the video and was shown from the waist up (Pi et al., 2022). All videos lasted 10–15 min.

### 3.4. Experimental Setting and Equipment

The experiment was conducted in a quiet, distraction-free laboratory. Before the sessions, the lighting was adjusted for viewing online instructional videos on a computer, and ambient noise was minimized. A fixed-height chair was used. Materials were displayed on a 23.1-inch Acer monitor with a resolution of 1920 × 1080 pixels. An EyeLink 1000 Plus eye tracker (SR Research Ltd., Ottawa, ON, Canada) and a portable Neuroscan Grael EEG system (Compumedics Neuroscan, Charlotte, NC, USA) simultaneously recorded physiological data.

Eye movements provide a dynamic measure of attentional allocation. Common indicators include time to first fixation, fixation duration, and transitions between areas of interest. The EyeLink 1000 Plus system used in this study (SR Research Ltd., Ottawa, ON, Canada) recorded monocular gaze at 1000 Hz with high tracking accuracy. A chinrest stabilized participants’ heads. Stimuli were presented on an external monitor connected to a Dell computer running Windows 7 Professional. The experiment was programmed in Experiment Builder. Eye-tracking data were exported in Data Viewer and analyzed in SPSS 27. Data quality was checked at the end of each calibration: a session proceeded only when the validation error was below 1° of visual angle at every calibration point, and calibration was repeated otherwise. Samples lost to blinks or to brief tracking failures were treated as missing and excluded from the computation of fixation duration; no interpolation was applied.

EEG is an important method for assessing cognitive effort and cognitive processing. EEG-based assessments may use event-related potentials, such as P300, or frequency-band features, including theta, alpha, beta, and gamma activity. EEG signals were recorded using a Neuroscan Grael amplifier (Compumedics Neuroscan, Charlotte, NC, USA) and 32 electrodes positioned according to the international 10–20 system. Bilateral mastoids (M1 and M2) served as reference electrodes, and REF and GND served as ground electrodes. EEG data were preprocessed in MATLAB (Version 9.14) using the EEGLAB toolbox (Delorme & Makeig, 2004). The raw data were high-pass filtered at 0.1 Hz and low-pass filtered at 50 Hz. Eye-movement artifacts (HEOG and VEOG) were removed by independent component analysis using the infomax algorithm, implemented with the runica function in EEGLAB. Visual inspection was then used to remove irregular noise such as muscle activity. Electrode–scalp impedance was kept below 10 kΩ at every site before recording began. Ocular components identified by independent component analysis were removed by component rejection rather than by segment deletion. The FCz electrode was designated as the site of interest before any analysis of the data was carried out, on the basis of the frontal-midline literature linking this site to cognitive effort, attentional control, and working-memory demand.

**Figure 2:**
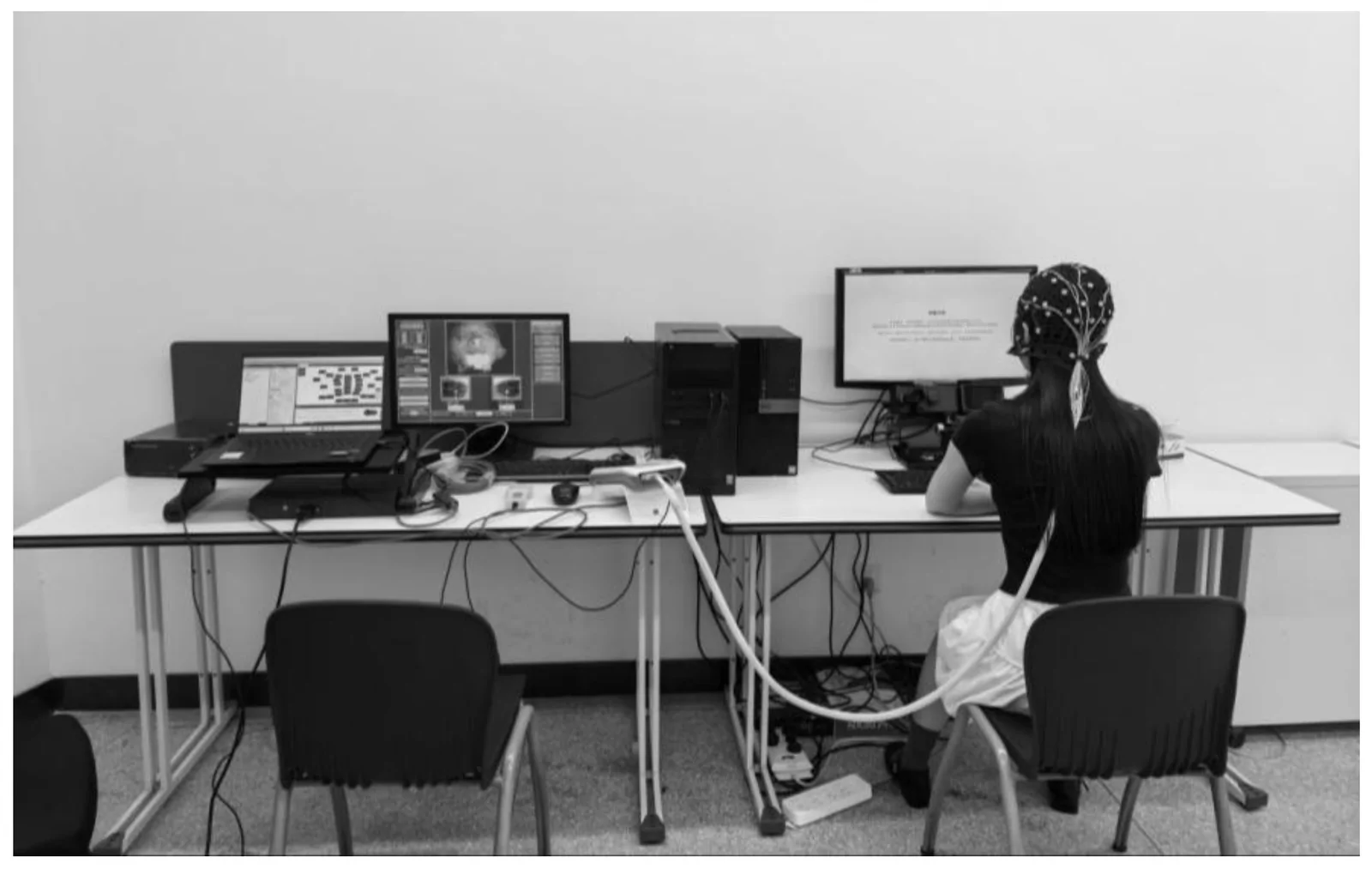
Experimental setting.

The experimental setting is shown in Figure 2. Participants wore an EEG cap and sat 65 cm from the computer monitor with their chin on the eye-tracker head support. They maintained this posture throughout the experiment and kept their head on the support to prevent data artifacts or loss caused by body movement.

### 3.5. Measures

Participants completed questionnaires before and after the experiment. The questionnaires covered six areas: demographics, prior knowledge, psychological distance, emotional attachment, learning satisfaction, and knowledge recall. The full research questionnaire is provided in Appendix A (Table A1). After a small-scale readability test, the final questionnaires were administered through the Wenjuanxing platform. The demographic section asked for participant number, gender, major and class, educational level, online-learning experience, and other basic information.

Prior knowledge was assessed with eight self-report items rated on a 7-point Likert scale (1 = no knowledge at all, 7 = extensive knowledge). Example items included “How well do you understand the functions and applications of hydroxyl radicals?” and “How well do you understand the codes used for shaded coat colors in British Shorthair cats?” The prior-knowledge test assessed familiarity with the video content and helped control for differences in participants’ existing knowledge.

Knowledge recall was assessed after the experiment. The test measured participants’ memory and mastery of the instructional content. Each video had 10 questions on key knowledge points: six single-choice questions, one multiple-choice question, and three true-or-false questions. Each correct answer received one point.

Psychological distance, emotional attachment, and learning satisfaction were measured on 7-point Likert scales. Participants indicated how well each statement described their experience (1 = does not describe me at all, 7 = describes me very well). The six-item psychological-distance scale was adapted from previous measures (Darke et al., 2016; Weidlich et al., 2024). An example item was “When I think about the virtual pedagogical agent used in the course, I feel psychologically close to it.” Items were reverse scored, so lower scores indicated less psychological distance. Cronbach’s alpha was 0.956. The four-item emotional-attachment scale was adapted from Jiménez and Voss (2014). An example item was “When I think about the virtual pedagogical agent used for this course topic, I feel a strong emotional bond with the course.” Higher scores indicated stronger emotional attachment. Cronbach’s alpha was 0.942. The five-item learning-satisfaction scale was based on prior educational research (Fryer et al., 2019). An example item was “Overall, I am satisfied with the course that used a virtual pedagogical agent for the current content.” Cronbach’s alpha was 0.952.

### 3.6. Experimental Procedure

The experiment comprised four stages. During preparation, the experimenter explained the study purpose, basic procedure, task requirements, and the use and precautions of the EEG and eye-tracking equipment. Participants were instructed to approach the video-viewing stage as an online class and to keep their head and body as still as possible. The experiment began after all questions had been answered.

EEG setup and pretest questionnaire. This stage lasted approximately 25 min. The experimenter seated the participant and fitted the EEG cap according to standard procedures. After connecting the cap to the amplifier and recording computer, the experimenter injected conductive gel into each electrode site with a syringe. During setup, participants scanned a QR code with their phone and completed the pretest questionnaire.

Eye-tracker calibration. Participants sat 65 cm from the monitor. The eye tracker was placed in front of the monitor, 55–60 cm from the participant’s eyes. Each participant placed their chin on a U-shaped rest to stabilize the head. The height of the rest was adjusted according to each participant’s height so that the eyes aligned with the upper third of the monitor. An image of the eyes was displayed on a nearby computer while the experimenter adjusted the camera angle. During calibration, participants fixated each calibration point until it disappeared and then located the next point.

Presentation of materials and posttest questionnaires. The experimental task comprised two sessions. In each session, participants watched one video and then scanned a code to complete the corresponding posttest questionnaire. The eye tracker and EEG equipment were calibrated before each session, and the session began only after stable recording was confirmed. Video order was randomly counterbalanced across participants to control for order effects. This stage lasted 30–40 min.

### 3.7. Data Processing and Analysis

Questionnaire data were exported from Wenjuanxing. Eye-tracking data were preprocessed in Data Viewer (Version 4.2.1) for the EyeLink 1000 Plus and exported as Excel files and heat maps. EEG data were recorded in Curry 8, preprocessed using the EEGLAB extension for MATLAB, and exported as electrode-power data. All data were organized in Excel. Descriptive statistics, repeated-measures analyses of variance, and mediation analyses were conducted in SPSS 27.0. Interaction terms were treated as the primary tests throughout, in line with the prediction that agent effects are conditional rather than additive. Effect sizes are reported as partial eta squared for the analyses of variance and as bootstrap confidence intervals for the conditional indirect effects. Given the sample size, these intervals are wide, and they are interpreted below as evidence about the direction of an effect rather than about its magnitude.

## 4. Results

Results are presented following the order of model construction. Behavioral results are reported first to verify whether the predicted agent-content interaction is observed for the primary outcomes (learning satisfaction and knowledge recall) and the two candidate socio-emotional mediators. The eye-tracking results follow, since they show whether the same interaction is visible in the allocation of overt attention during learning rather than only in retrospective appraisal. The electrophysiological results are reported next, as a further process-level check on the same interaction using a measure that does not depend on either report or gaze. The moderated mediation analyses come last, because the pathways they estimate presuppose that the preceding effects have been established. Section 4.5 then states, for each of the five hypotheses in turn, whether it was supported.

### 4.1. Behavioral Results

An independent-samples t test examined whether prior knowledge differed by instructional content type. The two content groups did not differ significantly in prior knowledge, *t* (38) = 0.565, *p* = 0.576.

Subsequently, descriptive statistics were calculated for all variables and experimental conditions. The results are shown in Table 1.

Repeated-measures analyses of variance were conducted to examine the effects of virtual pedagogical agent type and instructional content type on learning satisfaction, knowledge recall, psychological distance, and emotional attachment. For learning satisfaction, the main effect of agent type was significant, F(1, 38) = 12.72, *p* < 0.001, η_p_^2^ = 0.251. The main effect of instructional content type was not significant, F(1, 38) = 0.58, *p* = 0.450, η_p_^2^ = 0.015. The interaction was significant, F(1, 38) = 31.53, *p* < 0.001, η_p_^2^ = 0.453. Simple-effects tests showed that, with a human-like agent, satisfaction was significantly higher for traditional knowledge content (M = 4.95, SD = 1.16) than for interest-oriented popular science content (M = 3.24, SD = 1.42), *p* < 0.001. With a non-human agent, satisfaction was significantly higher for interest-oriented popular science content (M = 5.66, SD = 1.41) than for traditional knowledge content (M = 4.41, SD = 1.04), *p* = 0.003.

For knowledge recall, the main effect of agent type was significant, F(1, 38) = 9.78, *p* = 0.003, η_p_^2^ = 0.205. The main effect of instructional content type was also significant, F(1, 38) = 12.59, *p* = 0.001, η_p_^2^ = 0.249, as was the interaction, F(1, 38) = 5.31, *p* = 0.027, η_p_^2^ = 0.123. Simple-effects tests showed no significant difference between the two instructional content types with the human-like agent, *p* = 0.131. With the non-human agent, recall was significantly higher for interest-oriented popular science content (M = 9.35, SD = 1.57) than for traditional knowledge content (M = 7.25, SD = 1.83), *p* < 0.001.

For psychological distance, the main effect of agent type was not significant, F(1, 38) = 0.83, *p* = 0.368, η_p_^2^ = 0.021. The main effect of instructional content type was significant, F(1, 38) = 8.81, *p* = 0.005, η_p_^2^ = 0.188, as was the interaction, F(1, 38) = 43.97, *p* < 0.001, η_p_^2^ = 0.536. Simple-effects tests showed that, with the human-like agent, psychological distance was significantly lower for traditional knowledge content (M = 3.03, SD = 1.06) than for interest-oriented popular science content (M = 3.92, SD = 1.34), *p* = 0.024. With the non-human agent, psychological distance was significantly lower for interest-oriented popular science content (M = 2.43, SD = 1.16) than for traditional knowledge content (M = 5.01, SD = 1.32), *p* < 0.001.

For emotional attachment, the main effect of agent type was significant, F(1, 38) = 5.64, *p* = 0.023, η_p_^2^ = 0.119. The main effect of instructional content type was not significant, F(1, 38) = 0.08, *p* = 0.782, η_p_^2^ = 0.002. The interaction was significant, F(1, 38) = 28.02, *p* < 0.001, η_p_^2^ = 0.424. Simple-effects tests showed that, with the human-like agent, emotional attachment was significantly higher for traditional knowledge content (M = 4.54, SD = 1.18) than for interest-oriented popular science content (M = 2.93, SD = 1.32), *p* < 0.001. With the non-human agent, emotional attachment was significantly higher for interest-oriented popular science content (M = 5.13, SD = 1.53) than for traditional knowledge content (M = 3.70, SD = 1.53), *p* = 0.006.

### 4.2. Eye-Tracking Results

**Figure 3:**
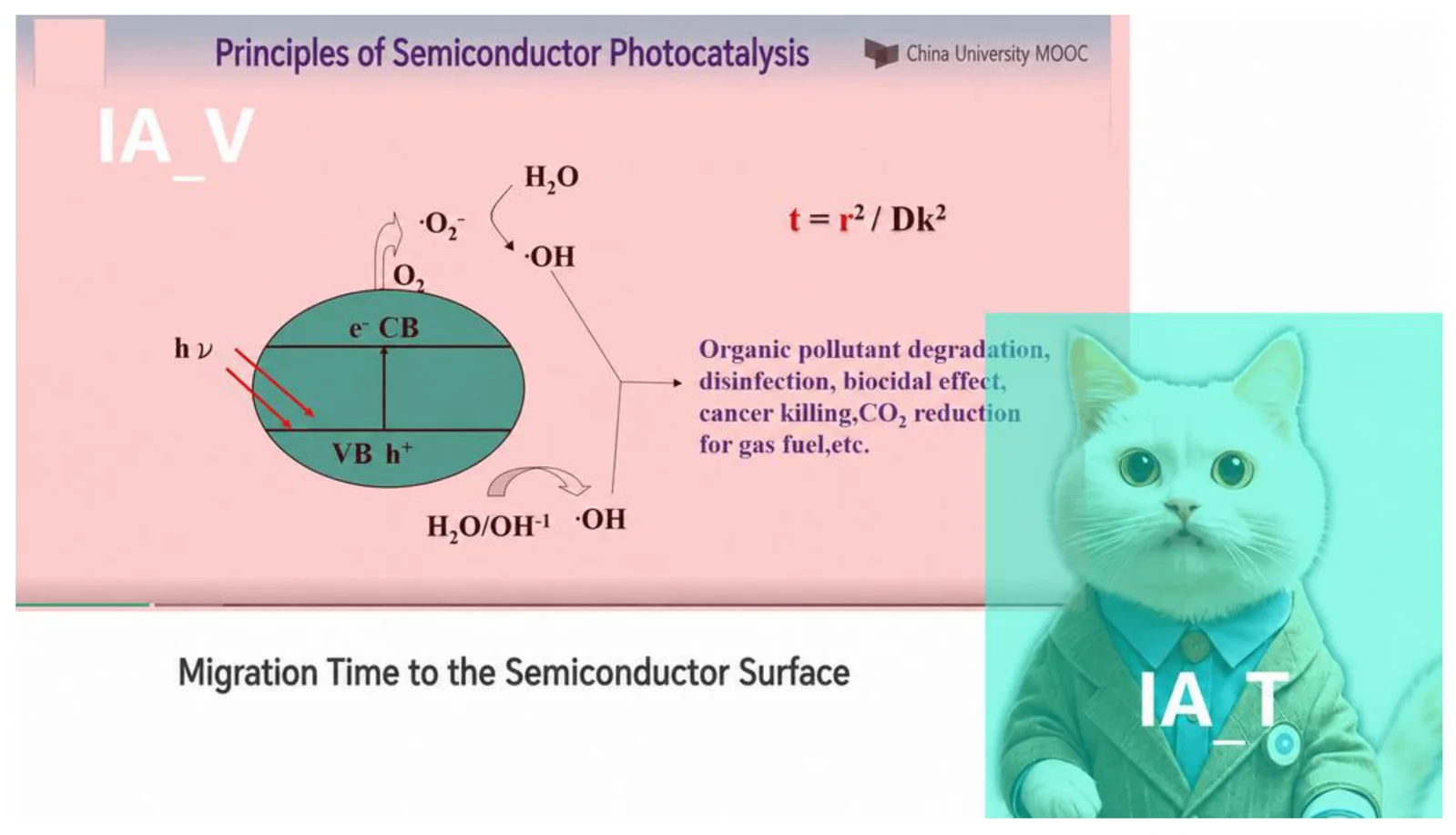
Definition of the areas of interest.

To examine how human-like and non-human agents affected attentional engagement across the two instructional content types, two areas of interest (AOIs) were defined: the course-video area (Interest Area_Video, IA_V) and the virtual-teacher area (Interest Area_Teacher, IA_T), as shown in Figure 3. Mean fixation duration and total fixation duration within each AOI were then summarized.

Repeated-measures analyses of variance examined attentional engagement across agent and content conditions, with fixation duration in the course-video AOI and the virtual-agent AOI as dependent variables (Table 2). For fixation duration in the course-video AOI, the main effect of agent type was significant, F(1, 38) = 11.97, *p* = 0.001, η_p_^2^ = 0.239. The main effect of instructional content type was not significant, F(1, 38) = 3.98, *p* = 0.053, η_p_^2^ = 0.095. The interaction was significant, F(1, 38) = 18.30, *p* < 0.001, η_p_^2^ = 0.325. Simple-effects tests showed that, with the human-like agent, fixation duration in IA_V was significantly shorter for traditional knowledge content (M = 207,018.20, SD = 85,877.66) than for interest-oriented popular science content (M = 303,523.20, SD = 63,590.35), *p* < 0.001. With the non-human agent, IA_V fixation duration did not differ significantly between interest-oriented popular science content (M = 293,530.30, SD = 59,807.38) and traditional knowledge content (M = 301,435.10, SD = 103,337.91), *p* = 0.769.

For fixation duration in the virtual-agent AOI, the main effect of agent type was significant, F(1, 38) = 4.31, *p* = 0.045, η_p_^2^ = 0.102. The main effect of instructional content type was also significant, F(1, 38) = 15.49, *p* < 0.001, η_p_^2^ = 0.290, as was the interaction, F(1, 38) = 4.32, *p* = 0.045, η_p_^2^ = 0.102. With the human-like agent, IA_T fixation duration was significantly longer for traditional knowledge content (M = 23,264.30, SD = 15,197.94) than for interest-oriented popular science content (M = 9369.80, SD = 5977.17), *p* < 0.001. With the non-human agent, IA_T fixation duration was also significantly longer for traditional knowledge content (M = 31,181.70, SD = 25,727.59) than for interest-oriented popular science content (M = 9368.90, SD = 6318.63), and the difference was larger, *p* < 0.001.

### 4.3. EEG Results

The FCz electrode in the frontocentral midline region was selected as an index of cognitive processing. FCz is closely associated with higher cognitive functions, including attentional control, cognitive effort, and working memory. Because the frequency bands are not equivalent indicators, spectral power was interpreted band by band rather than as a single, uniform index of processing depth. Frontal-midline theta power typically increases with cognitive effort and working-memory demand, and increases in beta and gamma power are commonly associated with active stimulus processing and information integration. Alpha power, by contrast, generally desynchronizes (decreases) when attention is directed toward external instructional material, so higher alpha is more often interpreted as reduced externally directed attention, or cortical idling, than as deeper processing, although alpha increases have also been linked to internally directed, elaborative processing. In the analyses reported below, the pattern of power across bands, rather than the absolute level of power in any single band, was therefore taken as the indicator of cognitive processing. Descriptive statistics for FCz power are shown in Table 3.

Analyses of variance similar to those used for the behavioral outcomes examined FCz power in the theta, alpha, beta, and gamma bands across experimental conditions. Agent type and instructional content type interacted significantly in every band: theta, F(1, 38) = 12.401, *p* < 0.001, η_p_^2^ = 0.246; alpha, F(1, 38) = 11.839, *p* < 0.001, η_p_^2^ = 0.238; beta, F(1, 38) = 11.105, *p* = 0.002, η_p_^2^ = 0.226; and gamma, F(1, 38) = 6.576, *p* = 0.014, η_p_^2^ = 0.148. Simple-effects tests showed no significant differences between traditional knowledge content and interest-oriented popular science content in any band with the human-like agent [F_Theta_(1, 38) = 1.289, *p* = 0.263, η_p_^2^ = 0.033; F_Alpha_(1, 38) = 1.345, *p* = 0.253, η_p_^2^ = 0.034; F_Beta_(1, 38) = 1.333, *p* = 0.256, η_p_^2^ = 0.034; F_Gamma_(1, 38) = 0.523, *p* = 0.474, η_p_^2^ = 0.014]. With the non-human agent, power was significantly higher for interest-oriented popular science content than for traditional knowledge content in every band [F_Theta_(1, 38) = 13.018, *p* < 0.001, η_p_^2^ = 0.255; F_Alpha_(1, 38) = 12.870, *p* < 0.001, η_p_^2^ = 0.253; F_Beta_(1, 38) = 12.569, *p* < 0.001, η_p_^2^ = 0.249; F_Gamma_(1, 38) = 7.206, *p* = 0.011, η_p_^2^ = 0.159]. Because power increased simultaneously in all four bands, including alpha, this pattern does not map onto a single band-specific mechanism and is therefore interpreted with caution in Section 5.3.

### 4.4. Moderated Mediation Analysis

To test the mediating roles of psychological distance, emotional attachment, and attentional engagement in the relationships between agent type and learning experience (learning satisfaction, knowledge recall, and cognitive processing), as well as the moderating role of instructional content type, the study followed the mediation procedure proposed by Zhao et al. (2010). Moderated mediation was tested using the models proposed by Hayes (2013), with 5000 bootstrap samples and 95% confidence intervals. Attentional engagement was represented by physiological eye-tracking data, and cognitive processing was indexed by EEG spectral power. Continuous variables were standardized, and categorical variables were dummy coded (0, 1) before analysis.

#### 4.4.1. Mediating Roles of Psychological Distance, Emotional Attachment, and Attentional Engagement Between Agent Type and Learning Satisfaction

Moderated mediation was tested with Model 7 in the SPSS PROCESS macro (Hayes, 2013). Agent type significantly and negatively predicted psychological distance (*b* = −0.85, *p* = 0.003) and significantly and positively predicted emotional attachment (*b* = 0.68, *p* = 0.033) and attentional engagement (*b* = 0.73, *p* < 0.001). Neither agent type nor attentional engagement significantly predicted learning satisfaction (*b* = 0.26, *p* = 0.135; *b* = −0.01, *p* = 0.937). Psychological distance significantly and negatively predicted learning satisfaction (*b* = −0.51, *p* < 0.001), whereas emotional attachment significantly and positively predicted learning satisfaction (*b* = 0.37, *p* = 0.009). The interaction between agent type and instructional content type significantly and negatively predicted psychological distance (*b* = −3.48, *p* < 0.001) and attentional engagement (*b* = −1.46, *p* = 0.003), and significantly and positively predicted emotional attachment (*b* = 3.04, *p* < 0.001). The results are shown in Table 4.

The moderated mediation analysis showed a significant conditional indirect effect through psychological distance, with instructional content type moderating the relationship between agent type and learning satisfaction, 95% CI [0.8938, 2.8055]. The corresponding conditional indirect effect through emotional attachment was also significant, 95% CI [0.3793, 2.1231] (Table 5). The conditional indirect effect through attentional engagement was not significant, 95% CI [−0.3381, 0.2599].

#### 4.4.2. Mediating Role of Psychological Distance Between Agent Type and FCz Spectral Power

Moderated mediation was tested with Model 7 in the SPSS PROCESS macro (Hayes, 2013). Agent type significantly and negatively predicted psychological distance (*b* = −0.85, *p* = 0.003), but did not significantly predict FCz Spectral Power (*b* = 0.13, *p* = 0.563). The interaction between agent type and instructional content type significantly and negatively predicted psychological distance (*b* = −3.48, *p* < 0.001). The moderated mediation analysis showed a significant conditional indirect effect through psychological distance, with instructional content type moderating the relationship between agent type and FCz spectral power, 95% CI [0.0589, 0.9183] (Table 5).

In addition, none of the mediation effects were significant when knowledge recall was the dependent variable.

### 4.5. Summary of Hypothesis Tests

H1, which predicted an effect of agent type on learning experience, was supported for learning satisfaction and for knowledge recall. The main effect is qualified by the interaction reported above, however, and should not be read as a general advantage of either agent. H2, which predicted mediation through psychological distance, was supported for learning satisfaction: the conditional indirect effect was significant in both content conditions and reversed in sign between them. H3, which predicted mediation through emotional attachment, was supported for learning satisfaction under interest-oriented popular science content, where the confidence interval excluded zero, but not under traditional knowledge content, where it did not. H4, which predicted mediation through attentional engagement, was not supported: agent type predicted fixation duration, but fixation duration did not predict satisfaction once the socioemotional mediators were included, and the conditional indirect effect was not significant. H5, which predicted moderation by instructional content type, was supported for learning satisfaction, knowledge recall, psychological distance, and emotional attachment, and the same interaction was present in the eye-tracking and electrophysiological data. Neither H2 nor H3 was supported when knowledge recall was the dependent variable.

## 5. Discussion

This study investigates how virtual pedagogical agents interact with instructional content to shape learners’ learning experiences, with psychological distance, emotional attachment, and attentional engagement serving as mediating variables, and reports several valuable findings.

### 5.1. Effects of Virtual Pedagogical Agents and Instructional Content on Learners’ Experience

Matching virtual pedagogical agents with instructional content increased learning satisfaction. Human-like agents were better matched with traditional knowledge content, whereas non-human agents were better matched with interest-oriented popular science content. The latter combination also improved knowledge recall. The match between a human-like agent and traditional knowledge content may allow social cues to elicit social presence and activate learners’ existing interpersonal schemas, thereby increasing involvement and emotional attachment. In multimedia learning, cognitive processing is jointly shaped by motivation, emotion, and metacognition (Moreno et al., 2001). Changes in emotion can therefore alter cognitive engagement and learning outcomes. The human appearance of a human-like agent may strengthen trust in the agent as an authoritative source of knowledge. For traditional knowledge content, this trust may reduce cognitive interference and help learners focus on the logic of the material. Polat et al. (2025) similarly found that human-like virtual instructors in knowledge-oriented videos were as effective as, or nearly as effective as, human instructors and improved perceived social presence and learning satisfaction. A non-human agent with interest-oriented popular science content may reflect the cognitive novelty of a non-human agent, which can stimulate curiosity. Hence, it meets the exploratory and entertaining demands of such content. Interest-oriented popular science requires conceptual discovery rather than authoritative transmission. A non-human agent may create productive cognitive tension and activate interest-driven learning, whereas a highly human-like agent may weaken the desire to explore. By reducing cues of interpersonal interaction, non-human agents may also direct greater attention to the information itself (Y. Zhang et al., 2025).

These results extend the literature reviewed in Section 2.2 in one respect and qualify it in another. They extend it by indicating that the comparisons on which much of that literature rests, agent versus no agent and human versus virtual instructor, are underdetermined: in this experiment the same non-human agent produced both the highest and among the lowest satisfaction scores, depending only on what it was used to teach. They qualify the conclusion of Polat et al. (2025) that human-like avatars perform comparably to human instructors, by suggesting that such equivalence may hold for the knowledge-oriented material typically used in those studies and need not extend beyond it. And they also sit awkwardly with accounts that treat human-likeness as a monotonic design variable, since the direction of the human-likeness effect reversed across content conditions here. What the data cannot establish is that this reversal is driven by the theoretical content distinction rather than by the particular topics and platforms used to instantiate it; that constraint is taken up in Section 5.4.

### 5.2. Mediating Roles of Psychological Distance and Emotional Attachment

A match between agent and content reduced psychological distance and strengthened emotional attachment, which in turn increased learning satisfaction. In other words, a good match fostered closeness and trust. This finding is consistent with previous research. Zhu (2024) showed that virtual characters can elicit genuine social responses: learners performed better on simple tasks when a character was present, without impaired performance on difficult tasks. The fit between a virtual instructor’s voice and the learning context can also increase credibility through social presence (Kim et al., 2022). This finding further suggests that alignment between instructor characteristics and instructional content helps establish trust and emotional connection. In immersive virtual learning environments, matching agent type with learning goals can improve the learning experience. Affective support from a virtual pedagogical agent also strengthens trust and attachment when it fits the user’s psychological needs (Yang & Oshio, 2025). Evidence from other fields supports the relationship between virtual agents and emotional attachment. Pantano and Scarpi (2022) found that intelligent social robots with strong reasoning capabilities elicited positive emotions and increased satisfaction. Singh (2022) found that anthropomorphism in intelligent assistants positively influenced human–machine emotional attachment and increased emotional connection and satisfaction. Coordinating agent-design features, including degree of anthropomorphism and social role, with the learning content is therefore an important pathway to stronger emotional connection and greater satisfaction. This conclusion is consistent with the anthropomorphic response framework proposed by Kim and Im (2023). They argued that anthropomorphic perceptions depend not only on appearance but also on an agent’s cognitive and emotional intelligence. When the agent’s displayed intelligence fits the task context, users more readily develop positive emotional responses and psychological closeness.

These results extend existing literature by locating psychological distance and emotional attachment as mediators rather than as outcomes, and by showing that the conditional indirect effects reverse in sign across content conditions, an asymmetry that a main-effects account of agent design cannot accommodate. They also depart from it in one respect: the two mediators did not behave alike. Psychological distance carried a significant indirect effect in both content conditions, whereas emotional attachment did so only under interest-oriented popular science content; the confidence interval for traditional knowledge content included zero. If this asymmetry replicates, it would suggest that reduced distance is the more robust route from agent design to satisfaction, and that attachment operates chiefly where the content itself invites an affective response. Neither mediator transmitted an effect onto knowledge recall, which indicates that the socioemotional pathway described by social agency theory reaches appraisal more readily than performance, a divergence between liking and learning that the agent literature has often noted but seldom modelled directly. Given the sample size, however, the contrast between a significant and a non-significant conditional indirect effect should not itself be treated as a reliable difference between the two mediators.

### 5.3. Eye-Tracking and Electrophysiological Evidence for Effects on Attentional Engagement and Cognitive Processing

The eye-tracking results showed that the interaction between agent type and instructional content type influenced attentional engagement. Traditional knowledge content, which is structured and factual, may depend more heavily on teacher explanation and guidance. Learners may look at the teacher to obtain information, understand complex concepts, or seek authoritative guidance, thereby allocating more cognitive resources to the teacher area. When a non-human agent presented interest-oriented popular science content, attention to the content area was moderate and attention to the teacher area was low. The results also suggest that visual cues from human-like agents, such as gestures, may distract learners. According to the cognitive theory of multimedia learning and CLT (Atkinson et al., 2005), excessive visual cues, such as simultaneous complex gestures and facial expressions, may compete for visual-channel resources. The resulting cognitive load may impair learning (Pi et al., 2026). Relative to the split-attention account (Ayres & Sweller, 2005), the present gaze data are only partly consistent. Learners looked longer at the agent under precisely the conditions in which satisfaction was lower, which is what a distraction account predicts, yet fixation on the agent did not predict satisfaction once psychological distance and emotional attachment were entered into the model.

In addition, what the electrophysiological data establish is narrower than what they invite. They show that FCz spectral power differed between content conditions under the non-human agent and did not differ under the human-like agent. Any account of why this occurred is inferential. One possibility is that the strong social cues of a human-like agent supply external structuring that makes processing demands more uniform across content types; another is that the non-human agent, offering fewer such cues, left learners to structure the material for themselves; a third is that the broadband difference reflects a general difference in signal amplitude rather than a cognitive process at all. Because power was recorded at a single midline electrode, these accounts cannot be distinguished with the present data, and no claim is made here about the recruitment of particular neural networks or executive systems. What carries weight is the convergence of the electrophysiological interaction with the behavioral and eye-tracking interactions, not the absolute magnitude of power in any band.

### 5.4. Limitations

This study has several limitations. First, no manipulation check assessed perceived fit. The high-fit conditions were defined from theory, so the study cannot confirm that learners perceived the two combinations as more congruent, and the interaction reported here is compatible with mechanisms other than perceived congruence. Therefore, “fit” in the present study denotes a theoretical interpretation of the interaction between agent and content rather than an experimentally demonstrated mechanism. Second, the sample was small, and the validity and generalizability of the findings require further examination. Third, the EEG analysis used frequency-band power from only one electrode. This measure cannot clearly distinguish the intrinsic, extraneous, and germane components of cognitive load or localize their neural sources. Additionally, notable individual differences exist in working-memory capacity, which may stem from neurodiversity, educational background, and other demographic characteristics and can exert potential influences on learners’ task performance. Fourth, and most consequentially for the scope of the conclusions, instructional content type was operationalised through two specific sets of materials that differ in topic, source platform, and production style as well as along the intended theoretical dimension. The results therefore provide evidence about the instructional conditions actually tested and cannot establish that human-like agents suit traditional knowledge content in general, or that non-human agents suit interest-oriented popular science content in general. A stronger design would hold topic constant while varying presentational framing, or would sample several topics within each category and treat topic as a random factor. Fifth, the two agents differed in voice as well as in appearance, and neither voice was pretested for naturalness or attractiveness, so the agent-type effect is a composite one whose components cannot be separated here. Finally, the sample was predominantly female and drawn from a single institution, which further constrains generalisability. Future studies should use larger and more balanced samples, longitudinal designs, and a broader set of materials within each content category. They should also measure perceived agent–content congruence directly, pretest agent voices, and record from full electrode arrays, so that the boundary conditions and processing mechanisms of the interaction between agent and content can be identified rather than inferred.

## 6. Conclusions

Integrating social agency theory and cognitive load theory, this study combined questionnaires, eye tracking, and EEG to examine how the interaction between virtual pedagogical agent type and instructional content type affects learning experience in online learning. A consistent interaction emerged. For learning satisfaction, psychological distance, and emotional attachment, human-like agents produced better outcomes with traditional knowledge content than with interest-oriented popular science content. Non-human agents showed the opposite pattern: interest-oriented popular science content produced greater satisfaction and emotional attachment and less psychological distance. Knowledge recall supported this pattern only for the non-human agent. Recall was significantly higher for interest-oriented popular science content than for traditional knowledge content, whereas recall did not differ by instructional content type with the human-like agent. The mediation analyses clarified how this interaction affected satisfaction. Psychological distance and emotional attachment produced significant moderated mediation effects between agent type and learning satisfaction. Thus, the agent-content combination first changed learners’ psychological distance from and emotional connection to the agent, which then affected satisfaction. This finding supports the socioemotional pathway proposed by social agency theory and indicates that the relation between agent-design features and instructional content, rather than either in isolation, is what bears on trust and psychological closeness. By contrast, the moderated mediation effect through attentional engagement was not significant. FCz spectral power also showed a significant interaction. With the human-like agent, power did not differ by instructional content type. With the non-human agent, FCz spectral power was significantly higher for interest-oriented popular science content than for traditional knowledge content in every frequency band. Because power rose simultaneously in all four bands, including alpha, this broadband pattern is not an unambiguous index of deeper processing and may partly reflect a general difference in signal amplitude. It is reported here as a difference in FCz spectral power that accompanied the behavioral interaction, and no stronger inference is drawn from it.

## Figures and Tables

**Table 1:** Descriptive statistics for all variables.

Virtual Agent Type	Instructional Content Type	N	Knowledge Recall		Learning Satisfaction		Psychological Distance		Emotional Attachment	
			M	SD	M	SD	M	SD	M	SD
Human-like agent	Traditional knowledge	20	7.00	1.52	4.95	1.16	3.03	1.06	4.54	1.18
	Interest-oriented popular science	20	7.70	1.34	3.24	1.42	3.92	1.34	2.93	1.32
Non-human agent	Traditional knowledge	20	7.25	1.83	4.41	1.04	5.01	1.32	3.70	1.53
	Interest-oriented popular science	20	9.35	1.57	5.66	1.41	2.43	1.16	5.13	1.53

Note: N = Number, M = Mean, SD = Standard Deviation.

**Table 2:** Fixation duration by experimental condition (ms).

Virtual Agent Type	Instructional Content Type	N	IA_V Fixation Duration		IA_T Fixation Duration	
			M	SD	M	SD
Human-like agent	Traditional knowledge	20	207,018.20	85,877.66	23,264.30	15,197.94
	Interest-oriented popular science	20	303,523.20	63,590.35	9369.80	5977.17
Non-human agent	Traditional knowledge	20	301,435.10	103,337.91	31,181.70	25,727.59
	Interest-oriented popular science	20	293,530.30	59,807.38	9368.90	6318.63

Note: N = Number, M = Mean, SD = Standard Deviation.

**Table 3:** Descriptive statistics for FCz spectral power.

Virtual Agent Type	Instructional Content Type	N	Theta		Alpha		Beta		Gamma	
			M	SD	M	SD	M	SD	M	SD
Human-like agent	Traditional knowledge	20	0.003	0.002	0.003	0.002	0.005	0.002	0.013	0.005
	Interest-oriented popular science	20	0.004	0.002	0.004	0.002	0.006	0.003	0.015	0.006
Non-human agent	Traditional knowledge	20	0.003	0.001	0.003	0.001	0.004	0.001	0.013	0.003
	Interest-oriented popular science	20	0.005	0.002	0.006	0.003	0.007	0.003	0.017	0.006

Note: N = Number, M = Mean, SD = Standard Deviation.

**Table 4:** Moderated mediation effects of agent type on learning satisfaction.

	Psychological Distance			Emotional Attachment			Attentional Engagement			Learning Satisfaction		
	*b*	*SE*	*t*	*b*	*SE*	*t*	*b*	*SE*	*t*	*b*	*SE*	*t*
Constant	3.60	0.14	26.27	4.07	0.16	26.03	0.01	0.10	0.01	4.87	0.72	6.76
Agent type	−0.85	0.27	−3.09	0.68	0.31	2.18	0.73	0.19	3.79	0.26	0.17	1.51
Instructional content type	0.24	0.27	0.87	−0.09	0.31	−0.30	−0.33	0.19	−1.74			
Agent type × instructional content type	−3.48	0.55	−6.34	3.04	0.63	4.85	−1.46	0.38	−3.81			
Psychological distance										−0.51	0.10	−5.06
Emotional attachment										0.37	0.09	3.91
Attentional engagement										−0.01	0.09	−0.08
*R* ^2^	0.40			0.27			0.30			0.81		
*F*	16.85			9.46			10.65			79.82		

**Table 5:** Moderated mediation effects.

Path	Effect	*SE*	95% Confidence Interval
Ind1: Agent type × instructional content type → psychological distance → learning satisfaction			
Overall effect	1.76	0.49	[0.8938, 2.8055]
Traditional knowledge content	−0.45	0.22	[−0.9494, −0.0629]
Interest-oriented popular science content	1.31	0.35	[0.6792, 2.0598]
Ind2: Agent type × instructional content type → emotional attachment → learning satisfaction			
Overall effect	1.13	0.45	[0.3793, 2.1231]
Traditional knowledge content	−0.31	0.20	[−0.7717, 0.0075]
Interest-oriented popular science content	0.82	0.32	[0.2765, 1.5022]
Ind3: Agent type × instructional content type → psychological distance → FCz spectral power			
Overall effect	0.47	0.22	[0.0589, 0.9183]
Traditional knowledge content	−0.12	0.08	[−0.3183, −0.0018]
Interest-oriented popular science content	0.35	0.16	[0.0471, 0.6561]

Note: Ind = indirect effect; *SE* = standard error.

## Data Availability

The data presented in this study are available on request from the corresponding author. The data are not publicly available due to privacy and ethical restrictions.

## Appendix A

**Table A1:** Research Questionnaire.

Variable	Items	Source(s)
Psychological Distance ^[R]^	When I think about the virtual pedagogical agent used in the course, I feel psychologically close to it. When I think about the physical characteristics of the virtual pedagogical agent used in the course, I can picture it concretely in my mind. When I think about the course and the functions of the virtual pedagogical agent it uses, they feel tangible to me. When I think about the course and the physical capabilities of the virtual pedagogical agent it uses, they seem very real to me. My online learning class may be enhanced by the virtual pedagogical agent used for the current course topic. The virtual pedagogical agent used for the current course topic will affect both me and students who share the same learning goals by enhancing our understanding of the course content.	(Darke et al., 2016; Weidlich et al., 2024)
Emotional Attachment	When I reflect on the virtual pedagogical agent used for the current course topic, I feel a strong emotional bond with the course. When I reflect on the virtual pedagogical agent used for the current course topic, I feel emotionally connected to the course. When I reflect on the virtual pedagogical agent used for the current course topic, I feel an affective connection with the course instructor. When I reflect on the virtual pedagogical agent used for the current course topic, I feel a strong sense of emotional attachment to the course instructor.	(Jiménez & Voss, 2014)
Learning Satisfaction	I greatly enjoy learning in this online course. The course, which uses a virtual pedagogical agent for the current course topic, is interesting. The course‘s use of a virtual pedagogical agent for the current course topic arouses my curiosity. Overall, I am satisfied with the course, which uses a virtual pedagogical agent for the current course topic. The course, which uses a virtual pedagogical agent for the current course topic, meets my needs as a learner.	(Fryer et al., 2019)

Note: Items marked ^[R]^ are reverse-coded.
